# A Domain Adaptation Meta-Relation Network for Knowledge Transfer from Human-Induced Faults to Natural Faults in Bearing Fault Diagnosis

**DOI:** 10.3390/s25072254

**Published:** 2025-04-03

**Authors:** Dong Sun, Xudong Yang, Hai Yang

**Affiliations:** School of Mechanical Engineering, Guizhou University, Guiyang 550025, China; 20130219@git.edu.cn (D.S.); haiy@uok.edu.gr (H.Y.)

**Keywords:** intelligent fault diagnosis, knowledge transfer, human-induced faults, natural faults, domain adaptation

## Abstract

Intelligent fault diagnosis of bearings is crucial to the safe operation and productivity of mechanical equipment, but it still faces the challenge of difficulty in acquiring real fault data in practical applications. Therefore, this paper proposes a domain adaptive meta-relation network (DAMRN) to achieve diagnostic knowledge transfer from laboratory-simulated faults (human-induced faults) to real scenario faults (natural faults) by fusing meta-learning and domain adaptation techniques. Specifically, firstly, through meta-task scenario training, DAMRN captures task-independent generic features from human-induced fault samples, which gives the model the ability to adapt quickly to the target domain tasks. Secondly, a domain adaptation strategy that complements each other with explicit alignment and implicit confrontation is set up to effectively reduce the domain discrepancy between human-induced faults and natural faults. Finally, this paper experimentally validates DAMRN in two cases (same-machine and cross-machine) of a human-induced fault to a natural fault, and DAMRN outperforms other methods with average accuracies as high as 99.62% and 96.38%, respectively. The success of DAMRN provides a viable solution for practical industrial applications of bearing fault diagnosis.

## 1. Introduction

In the modern manufacturing industry, bearings play a key role, which not only support mechanical transmission but also effectively reduce the friction between parts [1]. The stability and safety of bearings have a direct and important impact on the normal operation of mechanical equipment [2]. In view of this, the monitoring of the operating status of bearings and the study of fault detection are extremely important. Deep learning (DL) has been widely used in the field of bearing fault diagnosis and has become a popular direction in industrial research due to its ability to automatically extract feature signals, reduce the reliance on manual experience and powerful nonlinear modeling capabilities [3,4,5]. Numerous cutting-edge intelligent diagnostic techniques based on deep learning, such as the deep Boltzmann machine (DBM) [6], graph neural network (GNN) [7], and physical information neural network (PINN) [8], are emerging. These methods break through the limitations of traditional data-driven methods and significantly improve the accuracy and reliability of bearing fault identification. However, in the real world, it is not easy to obtain a large amount of suitable and real fault data due to the limitations of various factors, such as high cost of data collection, low and unpredictable probability of fault occurrence, and restricted access to data [9]. The results of many current studies are highly dependent on a large amount of failure data. In view of this, the results of the above studies do not have good applicability in the working environment of real machines [10].

In order to solve the problem of difficulty in obtaining a large number of fault samples, commonly used data generation or data augmentation methods include generative adversarial networks (GANs) and their variants [11], and diffusion models [12]. These methods help to improve model training by generating new data samples or augmenting existing data to improve the accuracy of fault diagnosis. Although GANs perform well in generating data, there may still be large differences between the distribution of generated data and the distribution of real data, resulting in the generated data not being able to replace fully real data for model training. The training process of diffusion models usually requires a large amount of computational resources and time, especially when generating high-quality and diverse data.

Some scholars have shifted their research focus to meta-learning, transfer learning, or the combination of meta-learning and transfer learning. Meta-learning, also known as “learning to learn”, aims at training situational tasks by using only a small amount of sample data so that it can quickly adapt to new tasks and has good generalization ability [13], thus showing a broad application prospect in many fields. Under the meta-learning paradigm, Zhang et al. [14] proposed a small-sample bearing fault diagnosis method based on model-agnostic meta-learning (MAML), which solves the problem of scarcity of fault samples in real industrial scenarios by means of a meta-learning strategy that optimizes model parameters. Li et al. [15] proposed a multiscale weighted integration model based on a light gradient boosting machine, which solves the problem of insufficient feature representation and weak model generalization in small-sample fault diagnosis by fusing multiscale features with a dynamic weight allocation mechanism. Wang et al. [16] proposed a small-sample bearing fault diagnosis method based on a self-embedding transformer, which solves the problem of traditional methods relying on manual feature engineering and poor model interpretability through adaptive feature embedding with an interpretable attention mechanism. More interestingly, Wang et al. [17] designed an interesting impulse neural network-oriented, brain-like learning algorithm based on the learning mechanism of a biological neural system and introduced a meta-learning strategy to apply it to the sample-scarce bearing fault diagnosis task. The core strength of transfer learning is its powerful ability to transfer knowledge. It is able to efficiently apply existing knowledge to new tasks even when data is scarce, and the source domain is significantly different from the target domain. This ability makes transfer learning show unique value when facing complex and changing real-world application scenarios [18]. Liang et al. [19] proposed an innovative diagnostic method based on a hybrid convolutional neural network model combined with an incremental migration learning strategy, which is able to adapt to different processing conditions. Experimental results show that this method increases the average accuracy rate substantially. Zhang et al. [20] applied the transfer learning strategy in the framework of a finite element integrated neural network, and not only verified its significant effect in improving computational efficiency but also confirmed the effectiveness of the strategy in many different scenarios, such as elasticity, elastoplastic, and multimaterials. In addition, some researchers have skillfully blended the strengths of meta-learning and transfer learning. Meta-learning is good at quickly adapting to new tasks, while transfer learning excels in knowledge transfer, and the combination of the two provides new ideas and methods for solving complex problems. This fusion strategy shows strong adaptability and efficiency when facing scenarios with scarce data, significant domain differences, and rapidly changing tasks. For example, the augmented meta-migration learning method proposed by Ma et al. [21] is able to achieve high-accuracy bearing fault diagnosis using a small amount of data under changing operating conditions. Experimental results show that the average accuracy of fault diagnosis reaches 95.2% after the introduction of augmented meta-migration learning. Zhong et al. [22] proposed an innovative cross-domain fault diagnosis method, which is based on the powerful ability of meta-learning to quickly adapt to new domains. On this basis, they further introduced a domain adaptation strategy, which effectively reduces the distribution difference between the source and target domains. Through this clever combination, the model is not only able to quickly learn the features of new tasks but also significantly improves the accuracy of cross-domain fault diagnosis, which provides new ideas and methods for solving complex and changing fault diagnosis problems.

Overall, meta-learning and transfer learning have indeed made significant progress in addressing the challenge of difficult access to fault samples. However, most of these advances rely on datasets of human-induced faults that have relatively standardized and controlled fault patterns. In contrast, faults in real industrial environments tend to be more complex, diverse, and unpredictable and differ significantly from artificially simulated faults. Although human-induced fault data can provide a basis for research to a certain extent, it is difficult to comprehensively cover the complexity of real faults due to their strong normality. Therefore, it cannot be directly applied to the actual industrial machine fault diagnosis. Nevertheless, the research direction of combining meta-learning with transfer learning still shows great potential and has achieved relatively leading results in related fields. However, to be truly applied to real industrial scenarios, further exploration is still needed to bridge the gap between human-induced faults and natural faults better.

Inspired by this, this paper hypothesizes that human-induced fault data contains some of the characteristics of natural faults. If the diagnostic knowledge from human-induced fault data can be used appropriately and effectively transferred to natural fault diagnosis, the challenge of obtaining expensive equipment fault data in real-world scenarios is expected to be significantly alleviated. Therefore, this paper proposes a domain-adaptive meta-relation network (DAMRN) for bearing fault diagnosis. DAMRN is a meta-learning framework designed for human-induced fault-to-natural fault knowledge transfer, aiming to solve the problem of cross-domain discrepancy between laboratory-simulated faults (human-induced faults) and real-scenario faults (natural faults) and, thus, to efficiently utilize the human-induced fault data in support of the diagnosis of natural faults. Specifically, through meta-task scenario training, DAMRN captures task-irrelevant general features from human-induced fault samples, enabling the model to adapt to target domain tasks quickly. Secondly, an explicit alignment and implicit adversarial complementary domain adaptation strategy is set, effectively reducing the domain discrepancy between human-induced faults and natural faults. This combination not only fully leverages the rapid adaptation capabilities of meta-learning to new tasks but also effectively transfers knowledge through domain adaptation techniques.

Compared with previous work, our contributions are as follows:

(1) This paper proposes a new solution path for human-induced fault to natural fault knowledge transfer, which is used to solve the problem of not being able to obtain a large number of fault samples for real-world precision devices.
(2) DAMRN is a meta-learning framework for cross-domain fault diagnosis, which incorporates multigranularity domain adaptation mechanisms, including explicit distributional alignment and implicit adversarial learning, based on the new task adaptation capability of meta-learning. The design aims to address the cross-domain robustness challenge under the new task.
(3) We demonstrate the feasibility of DAMRN with extensive experimental validation on two human-induced fault datasets and one natural fault dataset.

Next, the sections of this paper are set up as follows: Section 2 describes in detail the underlying theory of relational networks. Section 3 describes the implementation details of DAMRN in detail. Section 4 describes the experimental dataset and gives the experimental results with discussion. Section 5 concludes this work.

## 2. Meta-Relation Network

Meta-learning, also known as “learning to learn”, is centered on equipping models with the ability to adapt quickly to new tasks, much in the same way that humans learn [23]. For example, when children learn about a new animal, they can often recognize it accurately from a small number of pictures or videos rather than from a large number of samples. Traditional machine learning methods usually rely on a large number of data samples for model training, and learn the mapping relationship between features and labels directly, which is highly relevant. However, these models often need to be retrained when the application environment changes, which is particularly inconvenient when data collection is difficult. In contrast, meta-learning accumulates task experience by training on multiple tasks, thus enabling rapid adaptation when faced with new tasks. The more mainstream meta-learning models include the Siamese network [24], prototypical network [25], relation network [26], MAML [27], and matching network [28], etc. These models achieve fast learning and adaptation to new tasks with a small number of data samples through different mechanisms, such as learning the similarity between samples and optimizing the initialization parameters of the model.

**Figure 1:**
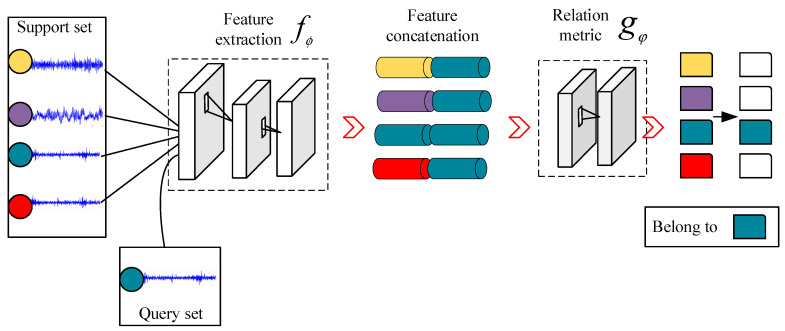
Relation network.

Before detailing the method proposed in this paper, it is necessary to briefly introduce the relation network because DAMRN is constructed based on the relation network. The structure of the relation network is shown in Figure 1, which is mainly composed of two parts: the feature extractor $f_{\emptyset }$ and the relation metric $g_{\varphi }$.

In the learning process of the relation network, the dataset is divided into support set $S={\left\{\left(x_{i}^{s},y_{i}^{s}\right)\right\}}_{i=1}^{n}$ and query set $Q={\left\{\left(x_{j}^{q},y_{j}^{q}\right)\right\}}_{j=1}^{m}$, where $x_{i}^{s}$ and $x_{j}^{q}$ denote the input samples, and $y_{i}^{s}$ and $y_{j}^{q}$ denote the category labels of the samples, respectively. The main role of the support set is to generate prototype features for each category; this process is achieved by superimposing all the sample features under the same category in the support set, i.e., the features of each category are summed element by element to obtain a prototype representation of the category. The query set is then used as a training sample to evaluate the performance of the model on new samples and update the model parameters accordingly. Specifically, the model predicts the category of a query sample by calculating the relationship scores between the features of the samples in the query set and the prototypical features of each category, i.e., it classifies the samples by learning the relationships between them. The process is described as follows: First, $f_{\emptyset }$ maps $x_{i}^{s}$ and $x_{j}^{q}$ to a uniform feature space to obtain $f_{\emptyset }(x_{i}^{s})$ and $f_{\emptyset }(x_{j}^{q})$. Then, the features of the support set samples and the query set samples are combined to obtain the combined feature $Z(f_{\emptyset }\left(x_{i}^{s}\right),f_{\emptyset }\left(x_{j}^{q}\right))$. Finally, $Z(f_{\emptyset }\left(x_{i}\right),f_{\emptyset }\left(x_{j}\right))$ is input into the relationship metric to calculate the relationship score $r_{i,j}$:

(1) $$r_{i,j}=g_{\varphi }\left(Z\left(f_{\emptyset }\left(x_{i}\right),f_{\emptyset }\left(x_{j}\right)\right)\right)$$

where Z is the concatenation operation.

Unlike Siamese networks, prototypical networks, and matching networks, these networks typically use a predefined fixed similarity metric function to measure the similarity between features. For example, Siamese networks extract features through subnetworks with shared weights and use the Euclidean distance metric function to compute the similarity between two samples. Prototypical networks are classified by computing the distance between the class prototype and the new samples, and commonly used distance metrics include the Euclidean distance and the cosine distance, among others. Matching networks, on the other hand, perform classification by calculating the similarity between the support set and the query set, usually using cosine similarity as the metric function. However, it is clear that these functions cannot cover all types of relationships. In this work [26], the authors demonstrate through a simple experiment that artificially designed metric functions tend to fail when dealing with nonlinear complex relationships. In contrast, relation networks use a convolutional neural network to train to obtain a learnable nonlinear similarity metric function that is able to measure different relationships more efficiently. This learnable similarity measure has enabled the relation network to achieve significant performance gains in few and zero-shot classification tasks, setting state-of-the-art records in several domains.

In addition, the *N*-way *K*-shot strategy of the relation network guides the model to learn task-independent generic feature representations so that it can better adapt to new tasks [29,30]. Therefore, in order to achieve knowledge migration from human-induced faults to natural faults, this paper proposes a DAMRN based on relation networks. In the next section, this paper introduces the specific structure and implementation of DAMRN in detail.

## 3. DAMRN

**Figure 2:**
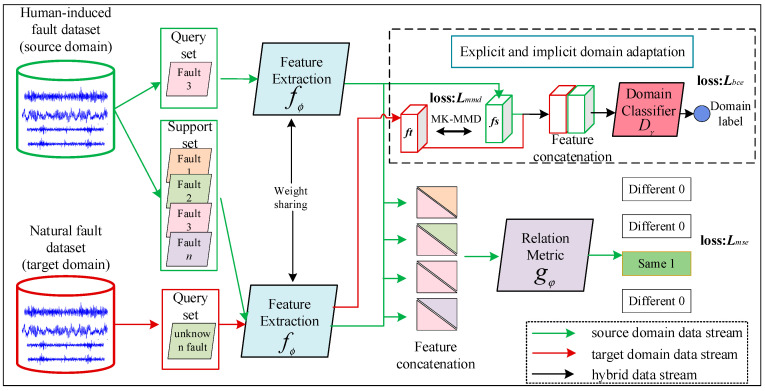
DAMRN framework.

DAMRN is a meta-learning framework designed for human-induced fault to natural fault knowledge transfer, aiming to solve the problem of cross-domain differences between laboratory simulated faults (human-induced faults) and real scenario faults (natural faults), as shown in Figure 2. DAMRN is based on a relation network and incorporates several domain adaptation mechanisms, including explicit distribution alignment and implicit alignment. The multi-kernel maximum mean discrepancy (MK-MMD) explicitly aligns the global feature distributions of the source and target domains. Implicit alignment, on the other hand, confuses the decision boundary of the domain discriminator through adversarial learning, forcing the model to generate domain-invariant features.

For explicit alignment and implicit alignment, we explain through an example: Explicit alignment is like using a ruler to measure the color distribution differences between two paintings and then adjusting the pigment ratios to make the overall tones consistent. Implicit alignment is like having two painters imitate each other’s styles, resulting in a convergence of painting styles, but the specific brushstroke details are formed through a game-like process. Mixed alignment uses both the ruler for quantitative adjustment (explicit) and allows the painters to compete dynamically (implicit), ultimately achieving a precise and natural style transfer.

**Figure 3:**
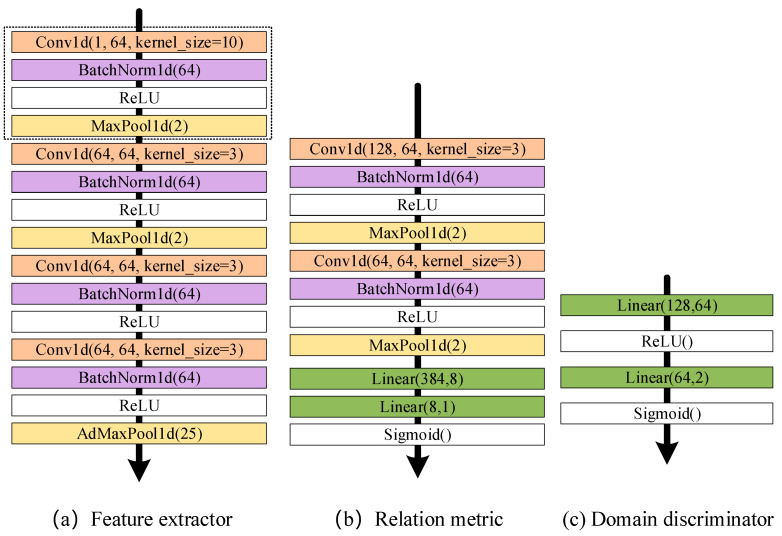
The detailed parameters of the feature extractor $f_{\emptyset }$, relation metric $g_{\varphi }$, and domain discriminator $D_{\gamma }$.

In addition, the detailed parameters of the feature extractor $f_{\emptyset }$, relation metric $g_{\varphi }$, and domain discriminator $D_{\gamma }$ of the DAMRN are shown in Figure 3.

### 3.1. Improvements to the N-Way K-Shot Strategy

The *N*-way *K*-shot is the core setting of few-shot learning, where *N*-way indicates that there are a total of *N* categories and *K*-shot indicates that each category contains *K* samples in the support set. The domain adaptation meta-relation network (DAMRN) retains the *N*-way *K*-shot learning strategy of relation networks, enabling rapid adaptation to cross-domain tasks through task simulation and relation metric. However, DAMRN has made significant improvements to its prototype generation mechanism, because the *K*-shot strategy of the original relation network generates category prototypes by element-by-element summation, as shown in Equation (2). We believe that this approach is too simple and crude because this linear stacking operation leads to a linear expansion of the range of values of the prototype features with the *K* value, which in turn affects the stability of the similarity calculation in the subsequent relationship module.

(2) $$p_{c}=\sum_{i=1}^{k}f_{\emptyset }\left(x_{i}^{s,c}\right)$$

where $p_{c}$ is the prototype of class c and $x_{i}^{s,c}$ denotes the *i*-th support sample of class *c*.

To address the above problems, this study proposes a hierarchical relation fusion strategy, where for each query sample $x_{j}^{q}$, its relation score with similar *K* support samples is computed independently, and finally, the K relation scores are averaged to represent the final relation score:

(3) $$r_{c,q_{j}}=\frac{\sum_{i=1}^{k}(g_{\varphi }\left(Z\left(f_{\emptyset }\left(x_{i}^{s,c}\right),f_{\emptyset }\left(x_{j}^{q}\right)\right)\right)}{k}$$

where $r_{c,q_{j}}$ denotes the average score of the query sample $x_{j}^{q}$ with class *c*.

Finally, the distance between the predicted relationship scores and the labels was calculated using the Mean Square Error (MSE) as a loss function:

(4) $$L_{mse}=\frac{1}{N_{q}\cdot C}\sum_{i=1}^{N_{q}}\sum_{c=1}^{C}{\left(r_{c,q_{j}}-y_{c,q_{j}}\right)}^{2}$$

where $N_{q}$ is the number of samples in the query set. *C* is the total number of categories. $y_{c,q_{j}}$ is the true label. $y_{c,q_{j}}=1$ if the query sample $x_{j}^{q}$ belongs to category *c*, and 0 otherwise.

### 3.2. Explicit Alignment

Explicit alignment reduces the difference between the feature distributions in the source and target domains directly through mathematical constraints, e.g., by using statistical metrics such as MMD, CORAL, etc., as loss functions. Explicit alignment forces the overall distribution of the two domains to be close, which has the advantage of being interpretable but may not be flexible enough for complex local differences [31].

In this paper, we have used MK-MMD to explicitly align the global distributions of features in the source and target domains, which is computed as follows:

(5) $$L_{mmd}=\sum_{k}{∥\frac{1}{n_{s}}\sum_{x_{s}\in D_{s}}\phi_{k}\left(x_{s}\right)-\frac{1}{n_{t}}\sum_{x_{t}\in D_{t}}\phi_{k}\left(x_{t}\right)∥}_{H_{k}}^{2}$$

where $\phi_{k}$ is the *k*-th kernel function, $x_{s}$ and $x_{t}$ denote the source and target domain features, respectively.

### 3.3. Implicit Alignment

For local nonlinear domain differences, DAMRN distinguishes features from source or target domains by a domain discriminator through the game idea of generative adversarial networks (GAN), while using the inverse gradient to optimize the feature extractor, forcing it to generate domain-invariant features. Implicit alignment has the ability of dynamic adaptation by adaptively adjusting the feature generation strategy through the adversarial signals of the discriminator. However, implicit adversarial relies on the quality of the discriminator and is prone to local optimum or over-optimization [31].

Finally, the domain classification loss is calculated using binary cross entropy:

(6) $$L_{d}=-\frac{1}{n}\sum_{i=1}^{n}\left[y_{i}\cdot logD_{\gamma }\left(x_{i}\right)+\left(1-y_{i}\right)\cdot log\left(1-D_{\gamma }\left(x_{i}\right)\right)\right]$$

where *n* is the value of Batch size and $y_{i}$ is the label of the domain, 0 or 1. $D_{\gamma }\left(x_{i}\right)$ is the predicted value.

### 3.4. Joint Optimization

During the training process, DAMRN learns task-independent generic feature representations [29,30] by meta-task simulation trained on the source domain; meanwhile, domain-aligned optimization of MMD and adversarial is performed using unlabeled data from the target domain to reduce the cross-domain distributional differences gradually. MMD provides a stable direction of distributional convergence for adversarial learning, while adversarial learning compensates for the lack of flexibility of MMD under complex local differences. Experiments show (see Section 4.4) that this explicit–implicit hybrid strategy can complement each other.

(7) $$L_{\mathrm{total}}=L_{mse}+\lambda_{1}L_{MMD}-\lambda_{2}L_{d}$$

where $\lambda_{1}$ and $\lambda_{2}$ are dynamic equilibrium coefficients. The updates of both $\lambda_{1}$ and $\lambda_{2}$ follow Equation (8) [32]:

(8) $$\lambda (e)=\frac{2}{1+e^{-10\cdot \frac{e}{Max\text{\_}e}}}-1$$

where *e* is the current number of training iterations, and *Max_e* is the total number of training iterations.

To facilitate the reader’s understanding of the learning process of DAMRN, the *N*-way *K*-shot and the definition of each input are simplified as follows: the source domain is divided into support set $S_{s}={\left\{\left(x_{s,i}^{s},y_{s,i}^{s}\right)\right\}}_{i=1}^{N*K}$ and $Q_{s}=\left(x_{s}^{q},y_{s}^{q}\right)$. The target domains are all used to make the query set $Q_{t}=(x_{t}^{q})$. Finally, the general learning process of DAMRN can be summarized as in Algorithm 1.
**Algorithm 1: The general learning process of DAMRN.**Input: $S_{s}={\left\{\left(x_{s,i}^{s},y_{s,i}^{s}\right)\right\}}_{i=1}^{N*K}$, $Q_{s}=\left(x_{s}^{q},y_{s}^{q}\right)$, $Q_{t}=(x_{t}^{q})$.for *i* in train epochs:   $f_{s}^{q}$, $f_{t}^{q}$ = $f_{\emptyset }\left(x_{s}^{q}\right),$ $f_{\emptyset }(x_{t}^{q})$  for $x_{s,i}^{s}$ in $S_{s}:$
    
$f_{s,i}^{s}$ = $f_{\emptyset }\left(x_{s,i}^{s}\right)$
    Calculate score $r_{c,q_{j}}$ for $Z\left(f_{s}^{q},f_{s,i}^{s}\right)$ using Equation (3).    Calculate $L_{mse}$ via Equation (4).  end for  Output total $L_{mse}$.  Calculate $L_{mmd}$ of $f_{s}^{q}$ and $f_{t}^{q}$ using Equation (5).  Domain prediction *pre1*, *pre2*
$=D_{\gamma }(f_{s}^{q})$, $D_{\gamma }(f_{t}^{q})$.  Calculate $L_{d}$ of *pre1* and *pre2* using Equation (6).  Backpropagation $L_{\mathrm{total}}=L_{mse}+\lambda_{1}L_{MMD}-\lambda_{2}L_{d}$.end for

## 4. Experimental Results and Discussion

### 4.1. Experimental Data

**Figure 4:**
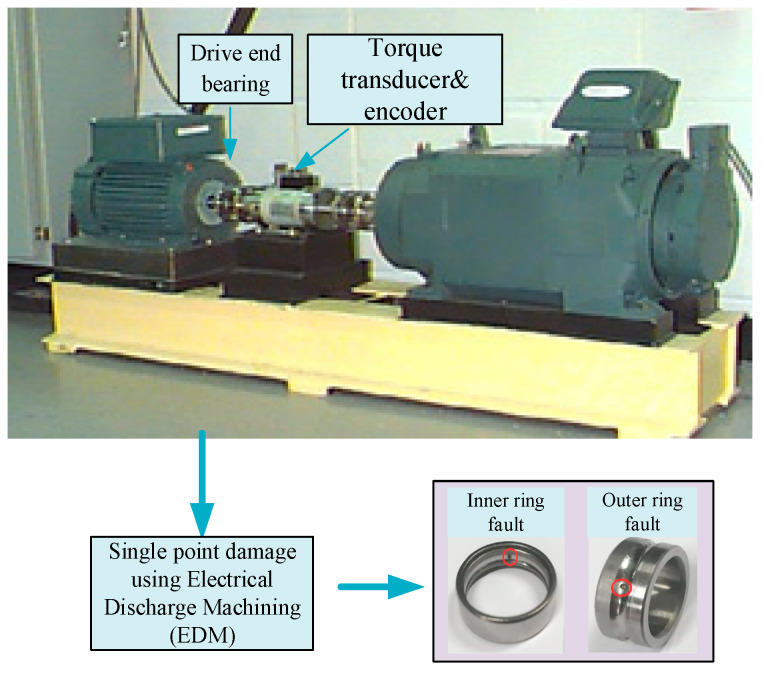
Bearing test bench of CWRU.

CWRU [33]: To validate the effectiveness of the DAMRN, this paper selected data from Case Western Reserve University (CWRU) as the source domain data, which is the easily collected human-induced fault dataset. This dataset is derived from a precision-designed bearing fault simulation platform, as shown in Figure 4. A single point of damage was simulated by EDM technology. Then, acceleration sensors are used to collect the vibration signals of the faulty bearings, and the sampling frequency is set to 12 kHz. Three types of samples are selected for this work, namely, inner-ring damage, outer-ring damage, and normal state, and the sample parameters are detailed in Table 1.

**Figure 5:**
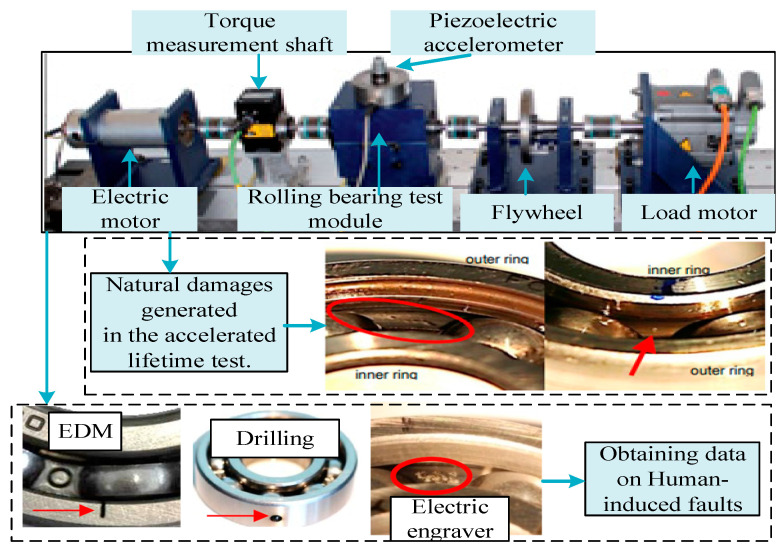
Bearing test bench of PU. This test stand collects vibration data from both human-induced faults and natural faults.

PU [34]: Natural faults were selected from the dataset provided by Paderborn University. The PU’s electromechanical drive system test platform (shown in Figure 5) consists of core components such as electric motors, torque sensing shafts, bearing test modules, inertial flywheels, and load motors. The platform uses deep groove ball bearings of type 6203 as test objects. The data on natural faults are obtained through accelerated life experiments. The data acquisition system synchronously records three-axis vibration signals, motor current signals, and mechanical parameters to form a multidimensional monitoring system, in which the vibration acceleration signals and motor current signals are sampled at a high frequency of 64 kHz, the mechanical parameters (radial load, rotational speed, and torque) are recorded at a frequency of 4 kHz, and the temperature monitoring is collected at a low frequency of 1 Hz. The study confirms that natural damage samples are more closely related to actual degradation mechanisms than human-induced fault samples [35], highlighting their unique value. The sample parameters are detailed in Table 2.

### 4.2. Experimental Details

#### 4.2.1. Sample Processing Details

**Figure 6:**
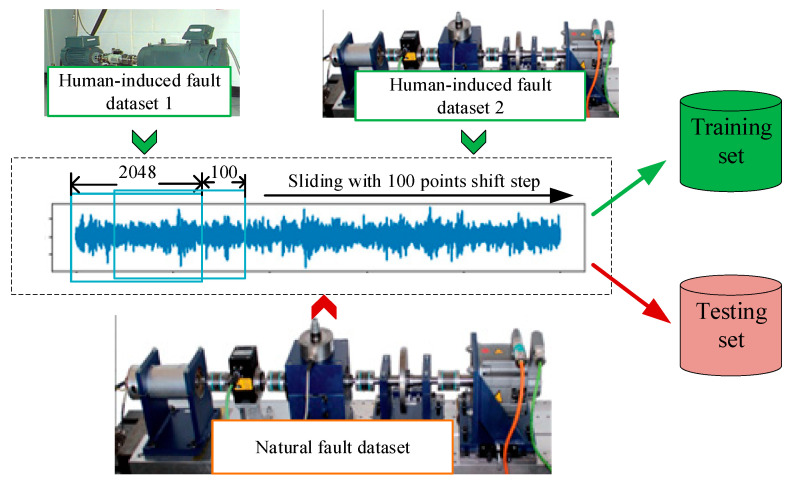
Training set and test set sample processing.

In the sample preparation stage, human-induced fault samples are used for training and natural fault samples are used for testing. Particularly worth mentioning is that because the data length is too short, this study refers to the related works [36,37,38] and introduces the sliding window resampling technique (the window step size is 100 data points, and the sampling length is 2048 points), which can obtain a large amount of data. The specific data processing flow is shown in Figure 6. The statistics of the number of training and testing samples are detailed in Table 3, where 700 samples of each class are randomly selected for the training set, totaling 2100 samples each, and 100 samples of each class are randomly selected for the testing set.

#### 4.2.2. Experimental Environment

In this study, a strictly controlled experimental environment is used to build the computational experimental platform, and the training system is constructed based on the NVIDIA GeForce RTX 3060 graphics processor and Pytorch 1.40 deep learning framework. In terms of hyper-parameter optimization, the optimal configuration is determined by grid search: the batch size is fixed at 64 to balance the memory consumption and gradient stability, and the learning rate is calibrated to 0.001 by pre-experiment. The model training period is 200 epochs to ensure that the model fully converges while avoiding the risk of overfitting. In order to enhance the statistical reliability of the experimental results, the experiments were repeated five times independently under each identical condition, and the average value was finally taken as the index for assessing the performance.

### 4.3. CASE1: Human-Induced Fault to Natural Fault Transfer in the Same Machine

**Figure 7:**
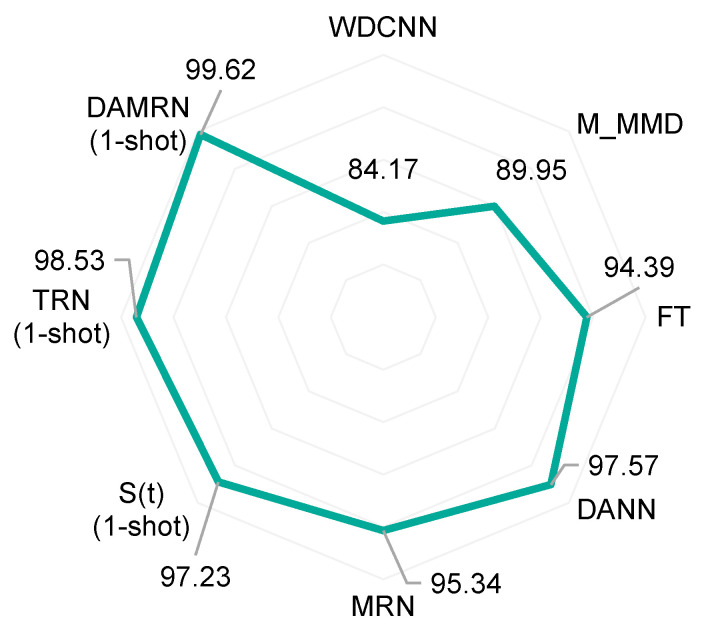
Experimental results on the transfer of human-induced faults to natural faults on the same machine.

In this section, we focus on the knowledge migration capability of DAMRN from human-induced faults to natural faults in the same machine environment, constructing a D → E migration scenario. In order to fully evaluate the performance of DAMRN, we compare it with a variety of other approaches, including the classical deep network model WDCNN, as well as the baseline models in the field of transfer learning, M_MMD [39], Fine-tuning (FT) [40], and DANN [32]. In addition, we introduced SOTA methods in the field of migration learning, such as MRN [41], S(t) [38], and TRN [42], to ensure that we can more accurately measure the strengths and weaknesses of DAMRN in knowledge migration tasks. All experiments were repeated five times, and the average classification accuracy (%) was used as the evaluation index; the specific results are shown in Figure 7.

From the experimental results in Figure 7, it can be seen that DAMRN achieves a classification accuracy of 99.62% in the D → E transfer scenario, which is higher than the other compared methods. This indicates that DAMRN has a clear advantage in the task of knowledge transfer from human-induced faults to natural faults in the same machine environment. This result shocked us because it is difficult to achieve this accuracy according to our previous work experience. We determined the reliability of this set of experimental results after several inspections as well as comparing the performance of the methods. Undoubtedly, the typical deep learning method WDCNN has the lowest accuracy (84.17%) among all methods and is 15.45% lower than DAMRN. This indicates that WDCNN is weak in adapting to the differences between the source and target domains when dealing with knowledge transfer tasks. The average classification accuracy of M_MMD is 89.95%, which is improved over WDCNN but still lower than other methods. This is because M_MMD relies only on the statistical alignment of multiple MMDs, which leads to alignment failure when the higher-order feature distributions of artificial and natural faults differ significantly. The average classification accuracy of FT was 94.39%, indicating the effectiveness of the fine-tuning approach in the knowledge transfer task. However, FT requires a small amount of target domain labelled data to operate effectively. DANN, MRN, S(t), and TRN also achieved good results.

Overall, DAMRN is significantly superior in knowledge transfer from human-induced faults to natural faults in the same machine environment. Its high accuracy indicates that DAMRN can effectively adapt to the differences between human-induced faults and natural faults for efficient knowledge transfer. In contrast, other methods such as DANN, MRN, S(t), and TRN, although showing some effectiveness in some aspects, are still lower than DAMRN in terms of overall performance.

### 4.4. CASE2: Human-Induced Fault to Natural Fault Transfer Across Machines

In real-world scenarios, high-end equipment such as aero-engine spindles and medical CT machine bearings are strictly prohibited from human damage to obtain fault data due to their importance and value. In view of this, this section focuses on knowledge transfer under different equipment conditions to validate the practical performance of the proposed approach in knowledge transfer from human-induced faults to natural faults across machines. Specifically, low-cost testbeds (such testbeds allow manual fault implantation) are used to construct source domain training sets. The diagnostic knowledge acquired from low-cost testbeds is migrated to high-value target devices by means of feature space mapping. This research direction is highly compatible with realistic conditions and not only has high research value but also faces large difficulty challenges. Three different sets of data, A, B, and C, shown in Table 1, are used as the source domain, and high-value device E, listed in Table 2, is used as the target domain. Four migration scenarios are constructed: single-source domain transfer (A → E/B → E/C → E) and multi-source domain joint transfer (ABC → E). The specific results are shown in Table 4, with bold representing the optimal performance.

From the experimental results in Table 4, it can be seen that DAMRN achieves optimal diagnostic accuracy (94.03–98.49%) in all transfer tasks, with an average improvement of 2.95 percentage points in average accuracy compared to the suboptimal method TRN. In addition, in single-source domain transfer scenarios (e.g., C → E), DAMRN (95.79%) improves by 24.59% over the traditional domain adaptation method, DANN (71.20%), which proves the effectiveness of DAMRN in mitigating feature distribution bias caused by the differences between different devices. The joint transfer of multiple source domains (ABC → E) resulted in a significant improvement in the performance of all methods, with DAMRN reaching a peak accuracy of 98.49%. This validates that the complementary nature of multiclass fault data enhances model generalization.

Compared to Case 1, some of the methods, such as WDCNN, FT, and DANN, show severe performance degradation, indicating the very high difficulty of cross-device transfer. However, the performance of DAMRN, TRN, and MRN still maintains high accuracy, indicating that their transfer strategies still keep running effectively in the more difficult scenarios.

### 4.5. Impact of K-Shot

**Figure 8:**
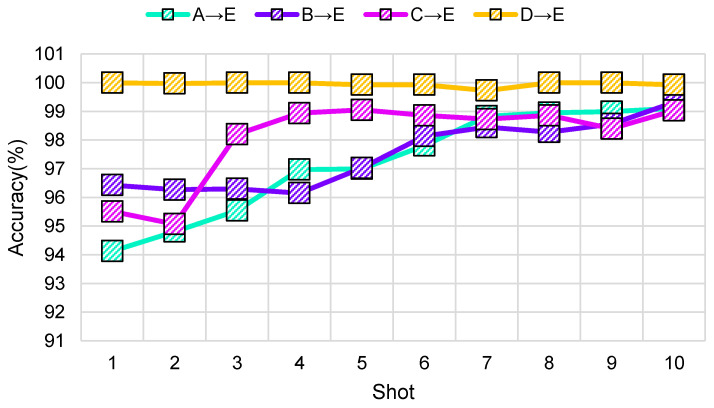
Impact of *K*-shot.

The learning process of DAMRN follows the meta-learning criterion of *N*-way *K*-shot. In order to fully evaluate the impact of *K*-shot on model performance, this paper designed a series of experiments to observe the performance of DAMRN in different tasks by varying the value of *K*. The experimental results are shown in Figure 8, which demonstrates in detail the average classification accuracy of DAMRN on each task (A → E, B → E, C → E, D → E) with different *K* settings.

As we know from Figure 8, the average classification accuracy of DAMRN on all tasks generally shows an increasing trend as the number of shots increases. This indicates that more samples in the support set help the model to measure the relationship between the source and target domains. Because the test samples are compared one by one with each of the *K* samples of each category in the support set, the average of the *K* scores of each category is taken, which avoids the effect of a single outlier sample. Specifically, the accuracy of D → F is consistently close to 100%. The accuracy of A → F gradually increases from 94.13% to 99.11% from 1 to 10-shot. The rest of the transfer tasks show similar improvements. However, from 6 to 10-shot, the accuracy of DAMRN tends to stabilize on all tasks, indicating that the model has already reached a high level of performance with a certain number of samples, and that further increase in the number of samples has a limited effect on the performance improvement.

### 4.6. Validation of the Effectiveness of the Transfer Strategy

The excellent knowledge transfer capability of DAMRN is mainly attributed to two key factors: firstly, the meta-learning paradigm of relation networks, which itself possesses a strong task-independent feature learning capability and can quickly adapt to new tasks; and secondly, the domain adaptation method we designed in DAMRN, which further enhances the performance of the model. To examine the designed domain adaptation method, this paper designed four sets of comparison experiments, as shown in Table 5.

**Figure 9:**
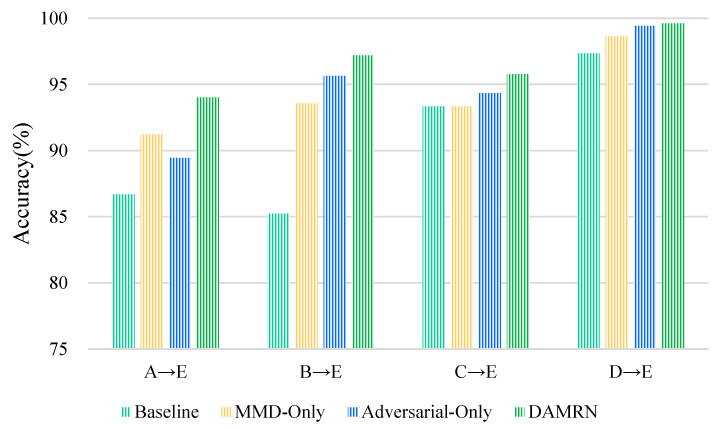
Performance of different variants.

See Figure 9. In all the tasks (A → E, B → E, C → E, D → E), the performance of the Baseline is relatively low, especially in the A → E and B → E tasks, where the gap with the optimal method is significant, suggesting that relying solely on the meta-learning framework cannot effectively overcome the problem of domain bias. Compared to the Baseline, MMD-Only improves its performance in all tasks. Specifically, MMD-Only improves significantly over Baseline in the A → E and B → E tasks but improves limitedly in the C → E task, even close to the Baseline level, reflecting that its explicit alignment relying on statistical distributions is insufficiently adaptive to some of the complex domain differences. Compared to MMD-Only, Adversarial-Only outperforms MMD-Only in the B → E and D → E tasks, suggesting that adversarial learning can dynamically capture the local features of domain differences; however, it is weaker than MMD-Only in the A → E task, probably due to the instability of adversarial training. DAMRN outperforms the other three sets of experiments in all tasks, showing that the simultaneous use of MMD and adversarial domain adaptation can reduce the discrepancy between the source and target domains more efficiently, validating the complementary nature of explicit and implicit alignment. In addition, for both explicit and implicit alignment, we use an example to describe it in Section 3, paragraph 2. 

### 4.7. Additional Discussion

If the DAMRN model can be successfully promoted and applied in practical industrial environments, it will bring a series of profound and positive impacts on the intelligent diagnosis and maintenance of machines. By utilizing real-time monitoring and warning functions, the downtime and maintenance costs caused by equipment failures can be effectively reduced. In real industrial applications, the computational requirements of the DAMRN model depend mainly on requirements, such as data volume and real-time performance. Taking the experimental hardware used in this study (detailed information is given in Section 4.2.2) as an example, the average time taken by DAMRN to diagnose a sample is about 2.09 × 10^−3^ s in this hardware configuration. Meanwhile, based on the sample acquisition method described in Section 4.2.1, the time to generate a sample to be tested for the two experimental platforms, CWRU (with a sampling rate of 12 kHz) and PU (with a sampling rate of 64 kHz), is about 8.34 × 10^−3^ and 1.56 × 10^−3^ s, respectively. Based on these time data, it can be seen that the DAMRN is able to meet the real-time requirements of the CWRU experimental platform; however, in the case of the high sampling rate of 64 kHz in the PU experimental platform, the problem of backlogged data streams will occur. In other words, for those industrial equipment monitoring scenarios with high requirements for both sampling rate and real-time, the performance requirements of the equipment running the DAMRN model will have to be further increased. However, for industrial equipment monitoring scenarios with lower sampling rates and real-time requirements, DAMRN can easily meet their needs. Overall, however, the application of compression techniques to the DAMRN model is necessary to adapt to the needs of different industrial applications better.

In addition, the maintenance of the model is the key to ensuring its long-term reliable operation. In practical applications, it is necessary to regularly monitor model performance, such as prediction accuracy, response time, etc., in order to identify and solve performance degradation problems in a timely manner. At the same time, with the accumulation of data and continuous changes in the business environment, it is necessary to update, optimize, and fine-tune the model based on the continuous learning framework to ensure that it always maintains the best operating state so that it can better adapt to the ever-changing application requirements. It must be clarified that current research focuses on specific fault types and datasets, which is mainly due to the preliminary nature of the research and the scope of available data. However, in order to widely apply the DAMRN model to practical industrial scenarios, it is necessary to conduct a comprehensive and in-depth evaluation of its performance on a wider range of workshop datasets and fault types.

## 5. Conclusions

This paper focuses on the difficulty of obtaining fault data in real scenarios and proposes a method called DAMRN. The method is designed to resolve the cross-domain differences between human-induced faults and natural faults in the laboratory and to support the diagnosis of natural faults with the help of human-induced fault data. DAMRN firstly captures task-independent generic features by virtue of an N-way K-shot learning paradigm, thus enabling fast adaptation to new tasks. Secondly, this paper has carefully designed the domain adaptation strategy of explicit and implicit mutual complementarity, which effectively reduces the domain differentiation between human-induced faults and natural faults. This paper validates this by launching extensive experiments on both datasets. The experimental results show that DAMRN has a high fault diagnosis accuracy of 99.62% in human-to-natural fault transfer for the same machine and a maximum diagnosis accuracy of 98.49% in human-to-natural fault transfer across machines. Compared with other methods, the performance is superior. In addition, this paper has fully validated the effectiveness of the DAMRN transfer strategy.

However, there is still some work that needs to be advanced, such as expanding to more diverse and fine-grained fault recognition, faster computation speed, and adaptability to actual industrial environments. In the future, we will further advance the related research work as far as conditions allow.

## Figures and Tables

**Table 1:** CWRU sample information.

Dataset Name	Fault Formation Mode	Name	Fault Location	Speed (rpm)
	Human-induced fault	0.021-Outer race	Outer ring	1772
A		0.021-Inner race	Inner ring	
		Normal	None	
	Human-induced fault	0.021-Outer race	Outer ring	1750
B		0.021-Inner race	Inner ring	
		Normal	None	
	Human-induced fault	0.021-Outer race	Outer ring	1730
C		0.021-Inner race	Inner ring	
		Normal	None	

**Table 2:** PU sample information.

Dataset Name	Name	Fault Formation Mode	Fault Location	Manufacturing Way
D	KI07	Human-induced fault	Inner ring	Electric engraver
	KA06		Outer ring	Electric engraver
	K004		Normal	None
E	KI04	Natural fault	Inner ring	Fatigue: pitting
	KA04		Outer ring	Fatigue: pitting
	K005		Normal	None

**Table 3:** Types and number of faults in training and test samples.

	Fault Location	Sample Size
Training	Inner	700
	Outer	700
	Normal	700
Testing	Inner	100
	Outer	100
	Normal	100

**Table 4:** Experimental results on the transfer of human-induced faults to natural faults across machines.

Transfer Tasks	WDCNN	M_MMD	FT	DANN	MRN (1-Shot)	S(t) (1-Shot)	TRN (1-Shot)	DAMRN (1-Shot)
A → E	44.87	69.47	76.4	68.36	86.12	91.22	90.15	**94.03**
B → E	43.80	74.40	80.47	67.29	84.23	90.29	93.53	**97.20**
C → E	40.20	69.87	75.2	71.20	92.63	89.56	94.64	**95.79**
ABC → E	48.46	76.46	82.33	68.73	93.45	92.87	95.38	**98.49**
AVG	44.33	71.25	77.36	68.90	89.11	90.99	93.43	**96.38**

**Table 5:** Different variants of DAMRN.

Variants	Domain Adaptive Method	Core Differences
Baseline	None	Retain only the meta-learning framework
MMD-Only	MMD domain adaptation	Explicit alignment of source and target domain feature distributions
Adversarial-Only	Adversarial domain adaptation	Implicitly aligning source and target domain feature distributions
DAMRN	Combination of MMD and adversarial domain adaptation	Explicit and implicit combination

## Data Availability

Data are available on request due to restrictions.

## References

[1] Ni Q., Ji J.C., Feng K., Zhang Y., Lin D., Zheng J. (2024). Data-Driven Bearing Health Management Using a Novel Multi-Scale Fused Feature and Gated Recurrent Unit. Reliab. Eng. Syst. Saf..

[2] Dong X., Zhang C., Liu H., Wang D., Chen Y., Wang T. (2025). A New Cross-Domain Bearing Fault Diagnosis Method with Few Samples Under Different Working Conditions. J. Manuf. Process..

[3] Tang J., Wei C., Li Q., Wang Y., Ding X., Huang W. (2022). Bearings Intelligent Fault Diagnosis by 1-D Adder Neural Networks. J. Dyn. Monit. Diagn..

[4] Liang X., Zhang M., Feng G., Xu Y., Zhen D., Gu F. (2023). A Novel Deep Model with Meta-Learning for Rolling Bearing Few-Shot Fault Diagnosis. J. Dyn. Monit. Diagn..

[5] Wang L., Zhao W. (2025). An Ensemble Deep Learning Network Based on 2D Convolutional Neural Network and 1D LSTM with Self-Attention for Bearing Fault Diagnosis. Appl. Soft Comput..

[6] Deng S., Cheng Z., Li C., Yao X., Chen Z., Sanchez R.-V. Rolling Bearing Fault Diagnosis Based on Deep Boltzmann Machines. Proceedings of the 2016 Prognostics and System Health Management Conference (PHM-Chengdu).

[7] Li Z., Li Y., Sun Q., Qi B. (2022). Bearing Fault Diagnosis Method Based on Convolutional Neural Network and Knowledge Graph. Entropy.

[8] Zhong J., Zheng Y., Ruan C., Chen L., Bao X., Lyu L. (2025). M-IPISincNet: An Explainable Multi-Source Physics-Informed Neural Network Based on Improved SincNet for Rolling Bearings Fault Diagnosis. Inf. Fusion.

[9] Zhang Y., Li S., Zhang A., An X. (2024). FW-UAV Fault Diagnosis Based on Knowledge Complementary Network Under Small Sample. Mech. Syst. Signal Process..

[10] Wang S., Wang D., Kong D., Wang J., Li W., Zhou S. (2020). Few-Shot Rolling Bearing Fault Diagnosis with Metric-Based Meta Learning. Sensors.

[11] Hu Z., Li Y., Han C. (2024). Transfer Learning Enabled Transformer-Based Generative Adversarial Networks for Modeling and Generating Terahertz Channels. Commun. Eng..

[12] Zhao Y., Sheng T., Li D. (2025). Data Augmentation Fault Diagnosis of Rolling Machinery Using Condition Denoising Diffusion Probabilistic Model and Improved CNN. IEEE Trans. Instrum. Meas..

[13] Ren Z., Zhu Y., Yan K., Chen K., Kang W., Yue Y., Gao D. (2020). A Novel Model with the Ability of Few-Shot Learning and Quick Updating for Intelligent Fault Diagnosis. Mech. Syst. Signal Process..

[14] Zhang S., Ye F., Wang B., Habetler T. (2021). Few-Shot Bearing Fault Diagnosis Based on Model-Agnostic Meta-Learning. IEEE Trans. Ind. Applicat..

[15] Li W., He J., Lin H., Huang R., He G., Chen Z. (2023). A LightGBM-Based Multiscale Weighted Ensemble Model for Few-Shot Fault Diagnosis. IEEE Trans. Instrum. Meas..

[16] Wang G., Liu D., Cui L. (2024). Auto-Embedding Transformer for Interpretable Few-Shot Fault Diagnosis of Rolling Bearings. IEEE Trans. Reliab..

[17] Wang J., Sun C., Nandi A.K., Yan R., Chen X. (2024). Brain-Inspired Meta-Learning for Few-Shot Bearing Fault Diagnosis. IEEE Trans. Neural Netw. Learn. Syst..

[18] Zhang S., Su L., Gu J., Li K., Zhou L., Pecht M. (2023). Rotating Machinery Fault Detection and Diagnosis Based on Deep Domain Adaptation: A Survey. Chin. J. Aeronaut..

[19] Liang Y., Wang Y., Li W., Pham D.T., Lu J. (2025). Adaptive Fault Diagnosis of Machining Processes Enabled by Hybrid Deep Learning and Incremental Transfer Learning. Comput. Ind..

[20] Zhang N., Xu K., Yin Z.-Y., Li K. (2025). Transfer Learning-Enhanced Finite Element-Integrated Neural Networks. Int. J. Mech. Sci..

[21] Ma L., Jiang B., Xiao L., Lu N. (2023). Digital Twin-Assisted Enhanced Meta-Transfer Learning for Rolling Bearing Fault Diagnosis. Mech. Syst. Signal Process..

[22] Zhong J., Gu K., Jiang H., Liang W., Zhong S. (2024). A Fine-Tuning Prototypical Network for Few-Shot Cross-Domain Fault Diagnosis. Meas. Sci. Technol..

[23] Feng Y., Chen J., Yang Z., Song X., Chang Y., He S., Xu E., Zhou Z. (2021). Similarity-Based Meta-Learning Network with Adversarial Domain Adaptation for Cross-Domain Fault Identification. Knowl.-Based Syst..

[24] Chicco D., Cartwright H. (2021). Siamese Neural Networks: An Overview. Artificial Neural Networks.

[25] Snell J., Swersky K., Zemel R. (2017). Prototypical Networks for Few-Shot Learning. Proceedings of the Advances in Neural Information Processing Systems.

[26] Sung F., Yang Y., Zhang L., Xiang T., Torr P.H.S., Hospedales T.M. (2018). Learning to Compare: Relation Network for Few-Shot Learning.

[27] Finn C., Abbeel P., Levine S. (2017). Model-Agnostic Meta-Learning for Fast Adaptation of Deep Networks. Assoc. Comput. Mach..

[28] Vinyals O., Blundell C., Lillicrap T., Kavukcuoglu K., Wierstra D. (2017). Matching Networks for One Shot Learning.

[29] Xue L., Jiang A., Zheng X., Qi Y., He L., Wang Y. (2023). Few-Shot Fault Diagnosis Based on an Attention-Weighted Relation Network. Entropy.

[30] Tang T., Qiu C., Yang T., Wang J., Zhao J., Chen M., Wu J., Wang L. (2023). A Novel Lightweight Relation Network for Cross-Domain Few-Shot Fault Diagnosis. Measurement.

[31] Zhang Y., Li S., He Q., Zhang A., Li C., Liao Z. (2023). An Intelligent Fault Detection Framework for FW-UAV Based on Hybrid Deep Domain Adaptation Networks and the Hampel Filter. Int. J. Intell. Syst..

[32] Ganin Y., Ustinova E., Ajakan H., Germain P., Larochelle H., Laviolette F., Marchand M., Lempitsky V., Csurka G. (2017). Domain-Adversarial Training of Neural Networks. Domain Adaptation in Computer Vision Applications.

[33] Case Western Reserve University Bearing Data Center Website | Case School of Engineering. https://engineering.case.edu/bearingdatacenter/welcome.

[34] Index of /Kat/BearingDataCenter. https://groups.uni-paderborn.de/kat/BearingDataCenter/.

[35] Lessmeier C., Kimotho J.K., Zimmer D., Sextro W. (2016). Condition Monitoring of Bearing Damage in Electromechanical Drive Systems by Using Motor Current Signals of Electric Motors: A Benchmark Data Set for Data-Driven Classification. PHM Soc. Eur. Conf..

[36] Zhang W., Peng G., Li C., Chen Y., Zhang Z. (2017). A New Deep Learning Model for Fault Diagnosis with Good Anti-Noise and Domain Adaptation Ability on Raw Vibration Signals. Sensors.

[37] Zhang A., Li S., Cui Y., Yang W., Dong R., Hu J. (2019). Limited Data Rolling Bearing Fault Diagnosis with Few-Shot Learning. IEEE Access.

[38] Zhang Y., Li S., Zhang A., Li C., Qiu L. (2022). A Novel Bearing Fault Diagnosis Method Based on Few-Shot Transfer Learning across Different Datasets. Entropy.

[39] Xiao D., Huang Y., Zhao L., Qin C., Shi H., Liu C. (2019). Domain Adaptive Motor Fault Diagnosis Using Deep Transfer Learning. IEEE Access.

[40] Li F., Chen J., Pan J., Pan T. (2020). Cross-Domain Learning in Rotating Machinery Fault Diagnosis Under Various Operating Conditions Based on Parameter Transfer. Meas. Sci. Technol..

[41] Wu J., Zhao Z., Sun C., Yan R., Chen X. (2020). Few-Shot Transfer Learning for Intelligent Fault Diagnosis of Machine. Measurement.

[42] Lu N., Hu H., Yin T., Lei Y., Wang S. (2022). Transfer Relation Network for Fault Diagnosis of Rotating Machinery with Small Data. IEEE Trans. Cybern..

